# Organized Sport Participation Is Associated with Higher Levels of Overall Health-Related Physical Activity in Children (CHAMPS Study-DK)

**DOI:** 10.1371/journal.pone.0134621

**Published:** 2015-08-11

**Authors:** Jeffrey J. Hebert, Niels C. Møller, Lars B. Andersen, Niels Wedderkopp

**Affiliations:** 1 School of Psychology and Exercise Science, Murdoch University, Murdoch, Western Australia, Australia; 2 Centre of Research in Childhood Health, Institute of Sports Science and Clinical Biomechanics, University of Southern Denmark, Odense, Denmark; 3 Department of Sport Medicine, Norwegian School of Sport Sciences, Oslo, Norway; 4 Sport medicine Clinic, the Orthopedic Department, Hospital of Middelfart, Institute of Regional Health Services Research, University of Southern Denmark, Middelfart, Denmark; Institute of Preventive Medicine, DENMARK

## Abstract

**Introduction:**

Many children fail to meet international guideline recommendations for health-related activity (≥60 minutes/day of moderate-to-vigorous physical activity [MVPA]), and intervention studies to date have reported negligible effects.

**Objective:**

Explore the associations of organized leisure-time sport participation with overall physical activity levels and health-related physical activity guideline concordance.

**Methods:**

This prospective cohort study was nested in the Childhood Health, Activity, and Motor Performance School Study Denmark. Study participants were a representative sample of 1124 primary school students. Organized leisure-time sport participation was reported via text messaging and physical activity was objectively measured over seven days with accelerometry. Associations between sport participation and physical activity level were explored with multilevel mixed-effects regression models and reported with beta coefficients (*b*) and adjusted odds ratios (aOR).

**Results:**

Participants were 53% female, with mean(SD) age = 8.4(1.4) years. Boys were more active than girls (*p*<0.001), and physical activity levels and guideline concordance decreased with age (*p*<0.001). Soccer participation at any frequency was associated with greater overall MVPA (*b*[95% CI] = 0.66[0.20,1.13] to 2.44[1.44,3.44]). Depending on participation frequency, this equates to 5–20 minutes more MVPA on the average day and 3 to 15 fold increased odds of achieving recommended levels of health-related physical activity (aOR[95%CI] = 3.04[1.49,6.19] to 14.49[1.97,106.56]). Similar associations were identified among children playing handball at least twice per week. Relationships with other sports (gymnastics, basketball, volleyball) were inconsistent.

**Conclusions:**

Many children, particularly girls and those in higher grade levels do not adhere to health-related physical activity recommendations. Organized leisure-time sport participation may be a viable strategy to increase overall health-related physical activity levels and international guideline concordance in children.

## Introduction

Physical inactivity (i.e., doing no or very little physical activity) is a leading cause of death worldwide, and the identification of strategies to increase physical activity levels in children is a public health priority [1]. Physical activity is essential for normal development in children [2] and important for the prevention of obesity and reduction of adiposity, particularly with respect to higher intensity activities performed over longer durations [3, 4]. Consequently, international guidelines suggest that children engage in at least 60 minutes of moderate to vigorous physical activity (MVPA) per day [3, 5]. However, many children fail to meet these minimum activity thresholds, and physical activity levels decline from childhood to adolescence [6, 7]. Recent systematic reviews examining the effectiveness of interventions to increase physical activity in children have reported only small treatment effects [8, 9]. Moreover, these reviews have urged cautious interpretation of past trial results owing to risk of bias and methodological limitations such as selection bias, lack of blinding, inconsistent outcome measurement, and failure to control for important confounders and clustering effects. Given the ramifications of physical inactivity in childhood, effective interventions are urgently needed.

Organized sport participation is one method with potential to increase overall physical activity levels in young people. Sports club membership predicts higher levels of leisure-time physical activity [10, 11], and organized sport participation increases the likelihood of meeting physical activity recommendations [11, 12]. Yet, other studies report that most time spent in organized sport involves sedentary to light intensity activity and sport participation alone does not ensure concordance with physical activity guidelines [13–15]. Moreover, increasing physical activity in one domain such as sport, may result in reduced activity at other times, thus attenuating the potential benefit of sport participation [16]. Consequently, the relationship between organized leisure-time sport participation and overall physical activity levels remains poorly understood and has been highlighted as a research priority [17].

The primary aim of this study was to explore for associations between sport participation and overall health-related physical activity level. Specifically, we examined for differences in average daily MVPA and concordance with physical activity recommendations among children who did and did not participate in different organized leisure-time sports at varying frequencies.

## Methods

### Study design

The current investigation was a prospective cohort study nested in the Childhood Health, Activity, and Motor Performance School Study Denmark (CHAMPS-study DK). This was a quasi-experimental trial designed to investigate the effects of increased physical activity performed by primary school students. All primary schools (N = 19) in the municipality of Svendborg, Denmark were invited to participate in the study. Ten schools, including six with augmented physical education programs and four control schools participated in the study. Additional details regarding the study sample, and procedures have been reported previously [18, 19]. The measures of physical activity were obtained during seven contiguous days, on two separate occasions. Ethical approval for this study was provided by the Regional Scientific Ethical Committee of Southern Denmark (ID S-20080047). All parents provided written consent and children provided verbal consent to participate prior to study enrolment.

### Study participants

First through sixth grade students from 10 public primary schools within the municipality of Svendborg, Denmark were enrolled in the study. Students from four schools received the traditional quantity of physical education (90 minutes/week), while students in the other schools received 270 minutes of physical education per week. The two school types were matched with respect to size and socioeconomic status and there were no differences in overall physical activity levels between the school types. Additional details of the study sample have been reported previously [19].

### Exposure to sport participation

Details of organized leisure-time sport participation over the previous week were reported by parents via mobile phone text messaging. Surrogate reporting by parents was undertaken due to concerns regarding the validity of self-reporting among children [20]. The text message reporting system involved a web-based method developed to monitor recurring events (SMS-Track, Esbjerg, Denmark). This approach is reliable, comparable to telephone interviews, and accepted by research participants [21].

At the end of each week, parents were asked to respond to the following question: How many times did [NAME OF CHILD] engage in organised sports during the last week?”. The parents were instructed to answer with a number between (0) and (8). An answer of (0) indicated that the child had not participated in organized leisure-time sport in the previous week. Answers (1) to (7) indicated the number of sport sessions during the week, while (8) indicated that the child had engaged in more than 7 sport sessions. When sport participation was reported, an additional question was sent, inquiring about the specific sport type(s).

All responses were automatically recorded into a database. In the case of an inappropriate response, parents were contacted by telephone for clarification. The average weekly response rate was 96.2%. Based on these reports, each participating child was categorized according to 1) sport participation status (yes or no), 2) sport type (e.g., soccer) and 3) the number of sport sessions. To help ensure adequate statistical power, we limited sport types to the five most prevalent sports: soccer, handball, basketball, gymnastics, basketball, and volleyball.

### Physical activity outcomes

Physical activity was assessed with the Actigraph GT3X accelerometer (Actigraph, Pensacola Florida) during 2 measurement periods (November to January and August to October) matched to the weeks of sport participation reporting. Trained research staff provided written and verbal instructions to the children and their parents and fit the accelerometers to each child’s right hip using customized elastic belts. The children were instructed to wear the device from the time they awoke until they went to bed for seven consecutive days; except when bathing or swimming.

A customized software program (Propero, version 1.0.18, University of Southern Denmark, Odense, Denmark) was used to process the accelerometry data. Physical activity data were recorded every 2 seconds and then collapsed into 10 second epochs. Digitalized, accelerometer signals were filtered with band limits of 0.25–2.5 HZ to help eliminate extraneous accelerations not associated with human movement (e.g., vibration). To distinguish inactivity from periods of non-wear, readings of zero activity lasting at least 30 consecutive minutes were interpreted as ‘accelerometer non-worn’. Physical activity data were included in the analysis if the child accumulated at least 10 hours of wear time on 4 or more days.

The physical activity outcomes were counts per minute and minutes spent in different intensities of physical activity. Counts per minute provide an estimate of overall mean physical activity throughout the average day. Additionally, time spent in sedentary, light, moderate, and vigorous physical activity intensities was estimated using established cut-points [22]. Finally, the average daily time spent at different physical activity intensities was used to categorize whether each child met the minimum level of health-related physical activity (at least 60 minutes of MVPA) recommended by international guidelines [5].

### Statistical analysis

Data management and statistical analyses were performed with Stata version 13.0 software (StataCorp, College Station, TX). Descriptive statistics stratified by sex, and grade level were calculated to explore the physical activity levels of all study participants.

To examine for differences in physical activity between children not participating in sport and those participating in specific organized sports, we generated multilevel mixed-effects regression models with random intercepts. The independent variable was sport participation and number of sport sessions per week. To identify the specific contribution of each sport to overall physical activity levels, children participating in multiple sports during the measurement period were excluded from the analyses. When we encountered cells containing less than 5 participants, the categories were collapsed across the number of sport sessions. Additional covariates comprising schools, classes, and individuals were entered as random effects, while sex, grade level, and school type were treated as fixed effects. Separate models were generated for each physical activity outcome variable. Unstandardized beta coefficients (*b*), representing the proportion of time per day spent in each physical activity intensity level were transformed to minutes by converting to percentages and multiplying by the mean daily accelerometer wear time.

Similar multilevel mixed-effects logistic regression models were used to examine for associations between sport participation and physical activity guideline concordance. The independent variable was sport participation classified by sport type and number of sport sessions per week. Dichotomous physical activity outcomes were defined by meeting an average threshold of at least 60 minutes of MVPA per day during the measurement period. Schools, classes, and individuals were entered as random effects, and sex, grade level, and school type were treated as fixed effects. As a preliminary step, we conducted a test for trend to identify differences in guideline concordance between children engaged in different sport types. When significant trends were identified, the model generated adjusted odds ratios (aOR) to quantify the association between sport participation and physical activity guideline concordance. Finally, two *post hoc* analyses were undertaken to explore for possible moderating, mediating, or confounding effects of 1) physical activity measurement period and 2) interactions between school type and sport participation. Alpha was 0.05 for all analyses.

## Results

**Fig 1:**
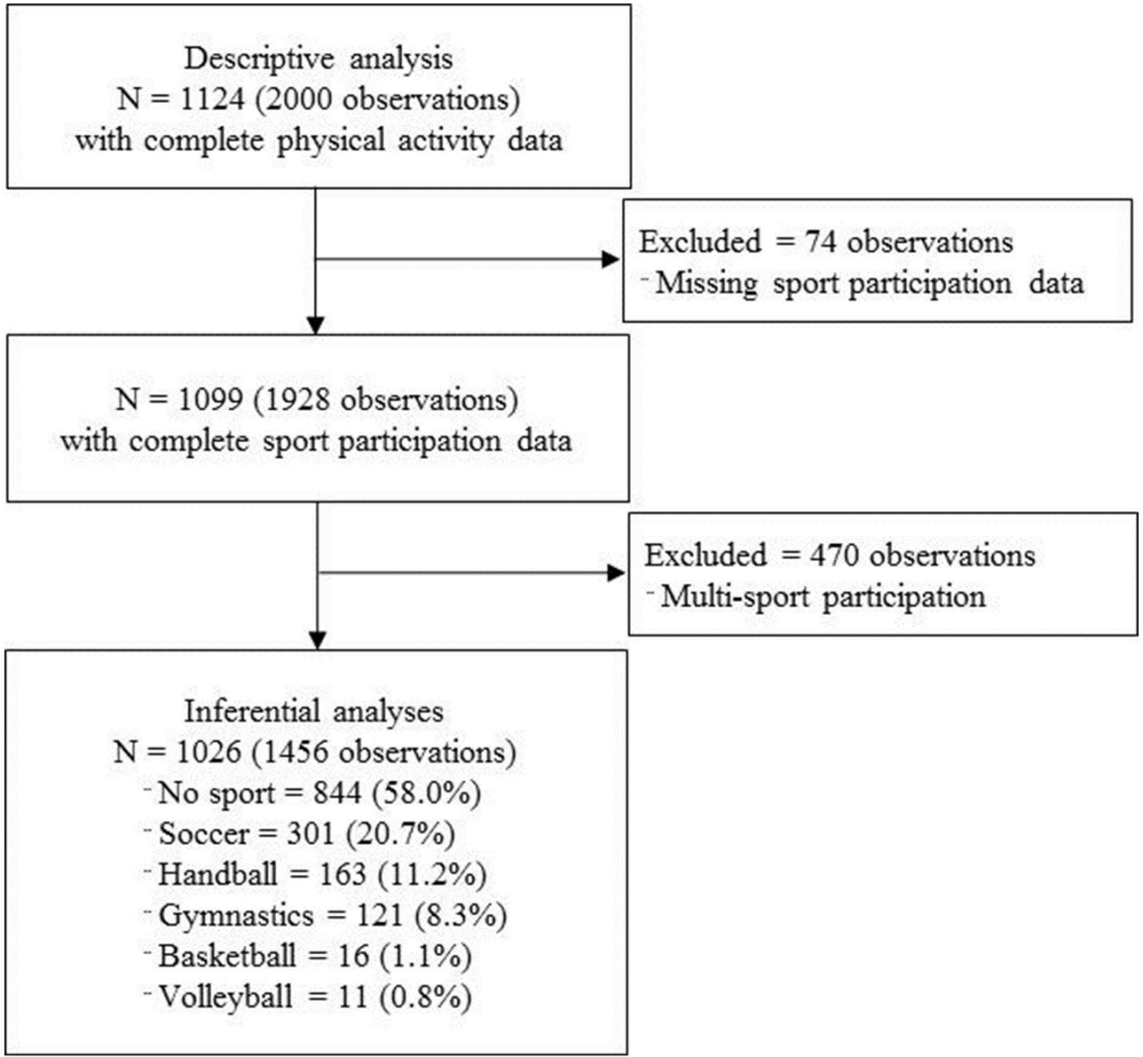
Study flow diagram.

Data from 1124 participants (53% female), comprising 2000 observations, were available and included in the descriptive analysis (Table 1, Fig 1). There were 1,015 and 985 observations in measurement rounds 1 and 2 respectively. The mean (SD) age at study enrolment was 8.4 (1.4) years and accelerometer wear time was 13.3 (0.66) hours.

**Table 1:** Descriptive physical activity outcomes stratified by sex and grade level.^a^

	Grade 1	Grade 2	Grade 3	Grade 4	Grade 5
Girls	n = 110	n = 119	n = 114	n = 123	n = 124
Wear time (h)	772.5(35.6)	791.5(35.0)	790.7(38.2)	808.4(37.7)	821.6(38.2)
Mean counts/min	621(139)	574(139)	529(128)	509(130)	469(123)
Sedentary (%)	58.5(4.9)	60.5(5.4)	62.5(5.3)	65.5(5.0)	67.5(5.1)
Light (%)	33.2(3.8)	31.8(4.2)	30.4(3.9)	27.6(3.7)	26.1(3.9)
Moderate (%)	5.4(1.3)	5.1(1.3)	4.6(1.3)	4.4(1.2)	4.1(1.1)
Vigorous (%)	2.9(1.2)	2.7(1.2)	2.4(1.1)	2.5(1.2)	2.3(1.1)
MVPA (%)	8.4(2.2)	7.7(2.3)	7.1(2.1)	7.0(2.1)	6.4(2.0)
MVPA (min/d)	66.6(17.3)	61.8(18.3)	56.3(17.0)	55.5(17.1)	51.1(16.0)
Guideline concordance	64.3%	50.0%	41.4%	37.8%	31.5%
Boys	n = 90	n = 105	n = 138	n = 99	n = 102
Wear time (h)	779.3(32.4)	789.1(35.1)	805.2(37.2)	809.3(38.7)	815.8(36.3)
Mean counts/min	687(140)	626(151)	604(135)	619(164)	586(176)
Sedentary (%)	57.2(4.8)	59.5(5.6)	61.7(5.5)	62.4(6.0)	63.9(6.4)
Light (%)	32.6(3.7)	31.2(4.0)	29.2(4.1)	28.2(4.2)	27.3(4.6)
Moderate (%)	6.6(1.3)	6.0(1.5)	5.8(1.4)	5.8(1.5)	5.4(1.7)
Vigorous (%)	3.7(1.4)	3.3(1.4)	3.3(1.3)	3.6(1.6)	3.4(1.6)
MVPA (%)	10.2(2.5)	9.3(2.7)	9.1(2.4)	9.5(2.9)	8.7(3.0)
MVPA (min/d)	81.8(19.8)	74.3(21.3)	72.3(19.4)	75.4(23.0)	69.7(24.1)
Guideline concordance	88.8%	76.2%	77.6%	71.8%	60.5%

^a^ Values are mean (SD) unless otherwise indicated.

Abbreviations: h, hours; MVPA, moderate to vigorous physical activity; min, minutes; d, day

Descriptive physical activity outcomes, stratified by sex and grade level, are presented in Table 1. The regression modelling indicated that boys spent more time engaged in MVPA than girls (*p* < 0.001) and identified a negative association between grade level and time in MVPA (*p* < 0.001). Similarly, boys were more likely than girls to meet recommended levels of physical activity (*p* < 0.001), and guideline concordance decreased with increasing grade level (*p* < 0.001).

The *post hoc* analyses identified no moderating, mediating, or confounding effects of measurement round or the interaction of school type and sport participation. There were significant interactions between school type and soccer participation in the models exploring the association between sports participation and overall physical activity level. However, this finding was inconsistent across differing participation frequencies and physical activity intensities and absent for other sports. Moreover, the inclusion of the interaction terms resulted in only minimal change to the parameter estimates with no difference to the inferential outcomes. Such findings were therefore considered to be spurious and the interactions were not included in the final models.

### Sport participation and physical activity

**Fig 2:**
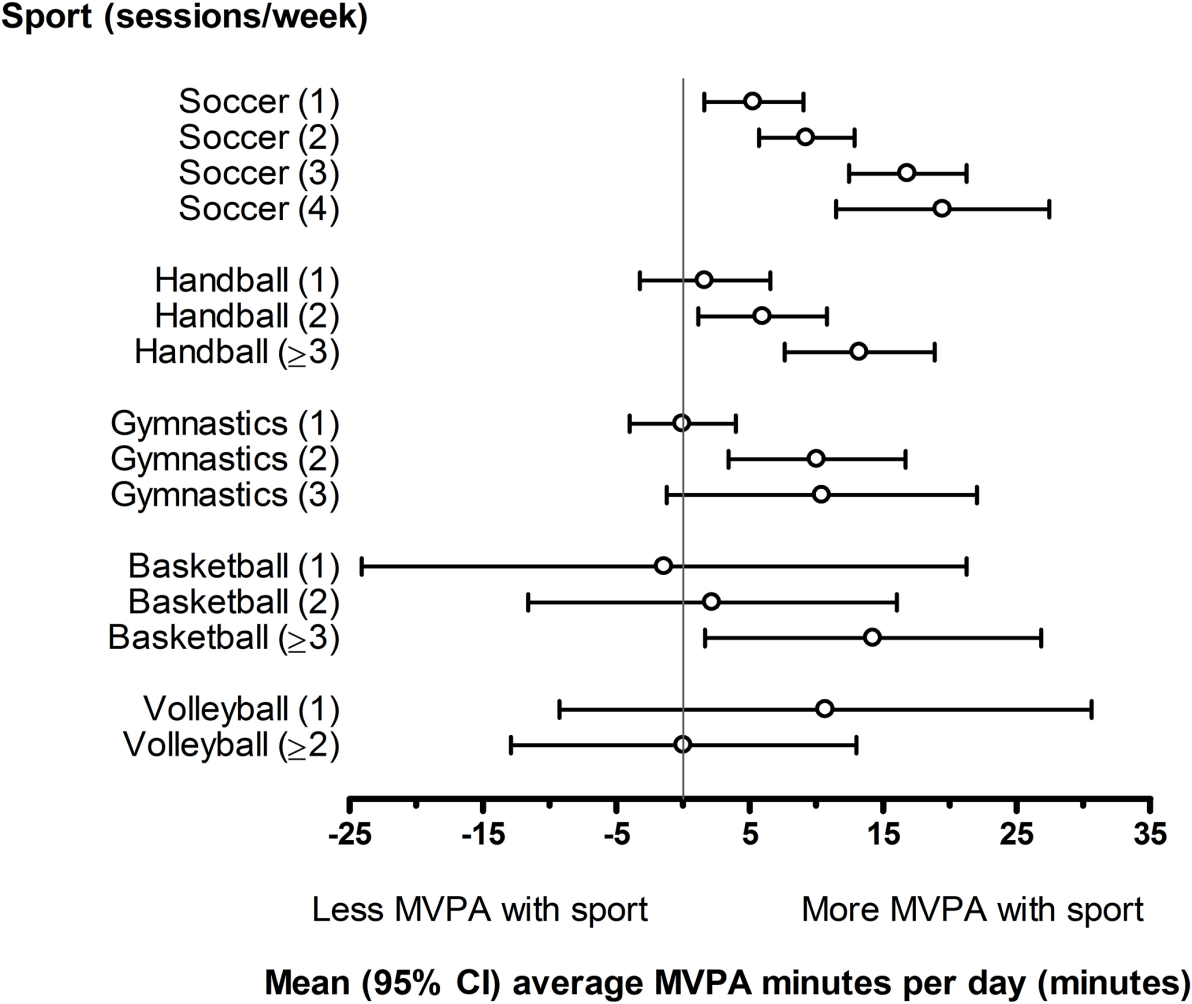
Average minutes of MVPA per day by sport type and participation frequency.

Table 2 reports the regression coefficients demonstrating the associations between sport participation at varying frequencies and the percentage of time per day spent in varying physical activity intensities, after controlling for sex, grade level, and school type. Soccer participation was associated with decreased time spent in sedentary activity (*b*[95% CI] = -1.08[-2.12,-0.03] to -4.54[-6.83,-2.24]), and increased time in moderate to vigorous physical activity (*b*[95% CI] = 0.66[0.20,1.13] to 2.44[1.44,3.44]). Depending on participation frequency, this equates to 9 (95% CI = 3,17) to 36 (95% CI = 18,55) minutes/day less sedentary time and 5 (95% CI = 2,9) to 20 (95% CI = 12,27) minutes more MVPA per day (Fig 2).

**Table 2:** Unstandardized beta coefficients (95% CI)^a^ between sport participation and average percent of daily time in physical activity.^b^

Sport (sessions/week)	Sedentary activity (%)	Light activity (%)	Moderate activity (%)	Vigorous activity (%)	MVPA (%)
Soccer (1)	**-1.08 (-2.12,-0.03)**	0.46 (-0.33,1.24)	**0.44 (0.17,0.70)**	0.23 (-0.01,0.48)	**0.66 (0.20,1.13)**
Soccer (2)	**-1.81 (-2.82,-0.80)**	0.66 (-0.10,1.41)	**0.55 (0.29,0.80)**	**0.62 (0.38,0.85)**	**1.16 (0.71,1.61)**
Soccer (3)	**-3.23 (-4.48,-1.99)**	**1.13 (0.20,2.07)**	**1.07 (0.76,1.39)**	**1.05 (0.76,1.34)**	**2.11 (1.56,2.66)**
Soccer (≥4)	**-4.54 (-6.83,-2.24)**	**2.14 (0.42,3.86)**	**1.03 (0.46,1.60)**	**1.42 (0.89,1.95)**	**2.44 (1.44,3.44)**
Handball (1)	-0.29 (-1.68,1.10)	0.11 (-0.93,1.15)	0.17 (-0.18,0.52)	0.04 (-0.28,0.36)	0.21 (-0.41,0.82)
Handball (2)	-1.30 (-2.67,0.07)	0.54 (-0.49,1.58)	**0.38 (0.04,0.73)**	**0.37 (0.05,0.69)**	**0.75 (0.14,1.35)**
Handball (≥3)	**-2.51 (-4.13,-0.90)**	0.81 (-0.41,2.03)	**0.76 (0.36,1.16)**	**0.91 (0.54,1.28)**	**1.66 (0.96,2.36)**
Gymnastics (1)	-0.23 (-1.34,0.89)	0.25 (-0.59,1.08)	0.01 (-0.27,0.30)	-0.02 (-0.28,0.25)	0.00 (-0.50,0.50)
Gymnastics (2)	**-2.48 (-4.37,-0.58)**	1.31 (-0.11,2.73)	0.38 (-0.10,0.85)	**0.88 (0.44,1.31)**	**1.26 (0.43,2.10)**
Gymnastics (3)	**-3.92 (-7.18,-0.67)**	**2.61 (0.19,5.03)**	0.21 (-0.63,1.04)	**1.08 (0.30,1.85)**	1.31 (-0.15,2.76)
Basketball (1)	0.49 (-5.81,6.80)	-0.40 (-5.08,4.28)	-0.19 (-1.82,1.44)	0.00 (-1.51,1.51)	-0.18 (-3.02,2.66)
Basketball (2)	0.56 (-3.42,4.55)	-0.98 (-3.99,2.03)	-0.54 (-1.53,0.45)	0.78 (-0.13,1.70)	0.28 (-1.46,2.01)
Basketball (≥3)	-1.22 (-4.78,2.34)	-0.54 (-3.19,2.12)	0.59 (-0.31,1.50)	**1.19 (0.35,2.03)**	**1.79 (0.21,3.37)**
Volleyball (1)	-1.79 (-7.50,3.92)	0.42 (-3.88,4.71)	0.72 (-0.71,2.14)	0.61 (-0.71,1.93)	1.34 (-1.16,3.84)
Volleyball (≥2)	-0.18 (-3.91,3.55)	0.39 (-2.43,3.21)	0.03 (-0.89,0.96)	-0.04 (-0.90,0.82)	0.01 (-1.62,1.63)

^a^ Adjusted for sex, grade level, school type

^b^ Bolded estimates indicate significant difference compared to children not participating in sport

Abbreviation: MVPA, moderate to vigorous physical activity

Participating in handball three or more times per week was associated with 20 (95% CI = 7,33) minutes less sedentary time per day on average (*b*[95% CI] = -2.51[-4.13,-0.90]).

Children playing handball two or more times per week engaged in 6 (95% CI = 1,11) (*b*[95% CI] = 0.75[0.14,1.35]) to 13 (95% CI = 8,19) minutes more MVPA per day (*b*[95% CI] = 1.66[0.96,2.36]). Associations between participation in other sports and physical activity levels were inconsistent (Table 2, Fig 2).

### Sport participation and physical activity guideline concordance

After controlling for sex, grade level, and school type, there was a significant trend for association (*p* < 0.001) between soccer or handball participation and physical activity guideline concordance (Table 3). Children playing soccer at any frequency demonstrated 3 to 15 times increased odds of achieving at least 60 minutes of MVPA per day as compared to children not participating in organized sport (aOR[95%CI] = 3.04[1.49,6.19] to 14.49[1.97,106.56]).

**Table 3:** Associations between sport participation and overall levels of MVPA (2009/2010).

Sport (sessions/week)	Odds ratio (95%CI)^a^	*p* value
Soccer (1)	**3.27** (1.56,6.85)	0.002
Soccer (2)	**3.04** (1.49,6.19)	0.002
Soccer (3)	**6.13** (2.31,16.23)	<0.001
Soccer (≥4)	**14.49** (1.97,106.56)	0.009
Handball (1)	1.07 (0.42,2.74)	0.878
Handball (2)	1.90 (0.78,4.62)	0.158
Handball (≥3)	**11.79** (3.58,38.84)	<0.001

^a^Adjusted for sex, grade level, school type

Bolded estimates are statistically significant

Abbreviation: MVPA, moderate to vigorous physical activity

Children participating in handball 3 or more per week were more likely (aOR[95%CI] = 11.79 [3.58,38.84]) to accumulate 60 minutes or more of MVPA per day compared to children not participating in sport. There were no significant trends for association between basketball, volleyball, or gymnastics participation and physical activity guideline concordance.

## Discussion

These study results have identified potentially important associations between organized leisure-time sport participation and overall health-related physical activity. Children participating in soccer were less sedentary, performed more MVPA, and were more likely to achieve recommended levels of physical activity compared to children not participating in organized leisure-time sport. Although participating in two or more sessions of handball per week was associated with increased MVPA, at least 3 sessions per week were necessary to demonstrate an association with international physical activity guideline concordance. Children participating in gymnastics, basketball, and volleyball were no more likely than children not engaged in sport to achieve recommended levels of health-related physical activity. The inconsistent outcomes associated with some sports may have resulted from type II error owing to low participation rates in those activities. Consistent with previous research [23], boys had higher levels of health-related physical activity than girls and physical activity decreased with increasing grade level.

Increasing physical activity levels in children has proven to be a challenging endeavour. A recent meta-analysis of clinical trials examining the effectiveness of interventions to increase physical activity levels in children reported only small to negligible treatment effects, equating to approximately 4 minutes of additional walking or running per day [9]. The current study results indicate that, organized leisure-time sport participation is associated with 5 to 20 minute increases in daily MVPA time and 3 to 15 fold increased odds of achieving physical activity guideline concordance. If these associations resulted from the effect of organized leisure-time sport participation, they would represent important increases in health-related physical activity. Consequently, organized leisure-time sport participation may be an effective strategy to increase health-related physical activity in children.

While movement performed during a sport session adds to overall activity levels, the nature of the relationship between sport participation and overall physical activity is unlikely to be limited to this direct contribution. This is consistent with our results demonstrating that children participating in just 1 session of soccer engaged in more daily MVPA over the course of the week and were 3 times more likely to meet physical activity recommendations. It is improbable that a single sport session alone would be sufficient to increase overall weekly health-related physical activity levels and raises the possibility that other factors modify or mediate these associations. For example, sport participation and physical activity in youth are both associated with improved psychosocial [24] and physical [25] health-related factors which may influence the relationship between sport and overall activity level.

To the casual observer, it may be a logical presumption that organized leisure-time sport participation results in measureable increases in overall health-related physical activity. Yet, previous evidence suggests that children and adults compensate for increasing physical activity levels in one domain (e.g., sport), by decreasing physical activity in other domains (e.g., leisure-time). This has been termed the activity-stat hypothesis [16], a topic of much debate and discussion in the physical activity arena. In the context of the current study, the activity-stat hypothesis would predict that children participating in organized leisure-time sport would compensate for their sport-related energy expenditures by decreasing their physical activity levels during other periods. Although the current study results were not consistent with the activity-stat hypothesis it is possible that children participating in organized leisure-time sport differed in ways not accounted for by the study design and analysis. While current evidence on the validity of the activity-stat hypothesis is inconsistent [26], future reporting aimed at accounting for methodological limitations of previous studies may inform this debate [27].

It is widely recognized that attaining recommended levels of physical activity is a central aspect of health and wellbeing. Healthcare providers have potential to make important contributions to the health behaviour of their patients, including physical activity practices. An essential step in this process is for clinicians and patients to measure and discuss physical activity and identify suboptimal behaviours. Some have argued that physical activity should be considered as the ‘fifth vital sign’ to be measured during every consultation, and practical recommendations toward this end for paediatric patients are available [28]. Measuring physical activity supports appropriate recommendations, counselling, and referral [29], and serves as a focus of the multinational ‘exercise is medicine’ global health initiative [30]. Our study results suggest that sport participation behaviour should be included as part of this process.

The current study had several strengths and limitations which inform the interpretation of these results. The major study strengths include that we objectively measured physical activity in a large cohort of children, using reliable and valid methods. Sport participation data were collected from parents using an approach that minimized the potential for recall bias, and response rates were very high. Additionally, our multilevel modelling accounted for several potential sources of confounding in the analyses. Inherent limitations of accelerometry include the inability to quantify movement during some activities (e.g., cycling and swimming), differential measurement accuracy, as well as the lack of widely accepted thresholds of activity intensity. As such, we were unable to measure all sources of physical activity performed by the participating children and it is possible that some activity was misclassified. The reporting of sport participation frequency did not include estimates of time per session and therefore the duration of activity may have differed between sport types. Thus, these limitations represent potential sources of residual confounding in our analysis. Prior to confident clinical or policy implementation, additional research is needed to further elucidate the causal pathway and mediating factors between sport and physical activity.

## Conclusions

Organized leisure-time sport participation was associated with increased health-related physical activity and concordance with international physical activity guideline recommendations for children. The strength of the associations varied depending on the type of sport and frequency of participation. Boys were more active than girls, physical activity levels declined with age, and many children did not attain the recommended levels of daily physical activity. These results highlight the potential importance of organized leisure-time sport participation as a strategy to increase overall health-related physical activity levels in children.

## References

[1] World Health Organization. Global health risks: mortality and burden of disease attributable to selected major risks Geneva, Switzerland: World Health Organization, 2009.

[2] Hills AP, King NA, Armstrong TP. The contribution of physical activity and sedentary behaviours to the growth and development of children and adolescents: implications for overweight and obesity. Sports medicine. 2007;37(6):533–45. Epub 2007/05/17. . 17503878 10.2165/00007256-200737060-00006

[3] Strong WB, Malina RM, Blimkie CJ, Daniels SR, Dishman RK, Gutin B, et al Evidence based physical activity for school-age youth. J Pediatr. 2005;146(6):732–7. Epub 2005/06/24. 10.1016/j.jpeds.2005.01.055 . 15973308

[4] Janssen I, Leblanc AG. Systematic review of the health benefits of physical activity and fitness in school-aged children and youth. The international journal of behavioral nutrition and physical activity. 2010;7:40 10.1186/1479-5868-7-40 20459784 PMC2885312

[5] World Health Organization. Global recommendations on physical activity for health. Geneva, Switzerland: World Health Organization, 2010 2010. Report No.: 9789241599979 9241599979.

[6] Troiano RP, Berrigan D, Dodd KW, Masse LC, Tilert T, McDowell M. Physical activity in the United States measured by accelerometer. Med Sci Sports Exerc. 2008;40(1):181–8. Epub 2007/12/20. 10.1249/mss.0b013e31815a51b3 . 18091006

[7] Nader PR, Bradley RH, Houts RM, McRitchie SL, O'Brien M. Moderate-to-vigorous physical activity from ages 9 to 15 years. JAMA. 2008;300(3):295–305. Epub 2008/07/18. 10.1001/jama.300.3.295 . 18632544

[8] Dobbins M, Husson H, DeCorby K, LaRocca RL. School-based physical activity programs for promoting physical activity and fitness in children and adolescents aged 6 to 18. Cochrane Database Syst Rev. 2013;2:CD007651. Epub 2013/03/02. 10.1002/14651858 CD007651.pub2. . 23450577 PMC7197501

[9] Metcalf B, Henley W, Wilkin T. Effectiveness of intervention on physical activity of children: systematic review and meta-analysis of controlled trials with objectively measured outcomes (EarlyBird 54). BMJ. 2012;345:e5888 Epub 2012/10/10. 10.1136/bmj.e5888 . 23044984

[10] Wichstrom L, von Soest T, Kvalem IL. Predictors of growth and decline in leisure time physical activity from adolescence to adulthood. Health psychology: official journal of the Division of Health Psychology, American Psychological Association. 2013;32(7):775–84. Epub 2012/08/29. 10.1037/a0029465 . 22924445

[11] Marques A, Ekelund U, Sardinha LB. Associations between organized sports participation and objectively measured physical activity, sedentary time and weight status in youth. J Sci Med Sport. 2015 10.1016/j.jsams.2015.02.007 . 25766508 PMC6235112

[12] Spinks A, Macpherson A, Bain C, McClure R. Determinants of sufficient daily activity in Australian primary school children. Journal of paediatrics and child health. 2006;42(11):674–9. Epub 2006/10/19. 10.1111/j.1440-1754.2006.00950.x . 17044893

[13] Guagliano JM, Rosenkranz RR, Kolt GS. Girls' physical activity levels during organized sports in Australia. Med Sci Sports Exerc. 2013;45(1):116–22. Epub 2012/07/31. 10.1249/MSS.0b013e31826a0a73 . 22843107

[14] Leek D, Carlson JA, Cain KL, Henrichon S, Rosenberg D, Patrick K, et al Physical activity during youth sports practices. Arch Pediatr Adolesc Med. 2011;165(4):294–9. Epub 2010/12/08. 10.1001/archpediatrics.2010.252 . 21135319

[15] Sacheck JM, Nelson T, Ficker L, Kafka T, Kuder J, Economos CD. Physical activity during soccer and its contribution to physical activity recommendations in normal weight and overweight children. Pediatr Exerc Sci. 2011;23(2):281–92. Epub 2011/06/03. . 21633140 10.1123/pes.23.2.281

[16] Rowland TW. The biological basis of physical activity. Med Sci Sports Exerc. 1998;30(3):392–9. Epub 1998/04/04. . 9526885 10.1097/00005768-199803000-00009

[17] Khan KM, Thompson AM, Blair SN, Sallis JF, Powell KE, Bull FC, et al Sport and exercise as contributors to the health of nations. The Lancet. 2012;380(9836):59–64. 10.1016/s0140-6736(12)60865-4 22770457

[18] Wedderkopp N, Jespersen E, Franz C, Klakk H, Heidemann M, Christiansen C, et al Study protocol. The Childhood Health, Activity, and Motor Performance School Study Denmark (The CHAMPS-study DK). BMC Pediatr. 2012;12:128 Epub 2012/08/22. 10.1186/1471-2431-12-128 22906115 PMC3483192

[19] Moller N, Tarp J, Kamelarczyk E, Brond J, Klakk H, Wedderkopp N. Do extra compulsory physical education lessons mean more physically active children—findings from the childhood health, activity, and motor performance school study Denmark (The CHAMPS-study DK). The international journal of behavioral nutrition and physical activity. 2014;11(1):121 Epub 2014/09/25. 10.1186/s12966-014-0121-0 . 25248973 PMC4180151

[20] Baranowski T, Smith M, Baranowski J, Wang DT, Doyle C, Lin LS, et al Low validity of a seven-item fruit and vegetable food frequency questionnaire among third-grade students. Journal of the American Dietetic Association. 1997;97(1):66–8. Epub 1997/01/01. 10.1016/S0002-8223(97)00022-9 . 8990421

[21] Johansen B, Wedderkopp N. Comparison between data obtained through real-time data capture by SMS and a retrospective telephone interview. Chiropr Osteopat. 2010;18:10. Epub 2010/05/27. doi: 1746-1340-18-10 [pii] 10.1186/1746-1340-18-10 20500900 PMC2883994

[22] Evenson KR, Catellier DJ, Gill K, Ondrak KS, McMurray RG. Calibration of two objective measures of physical activity for children. J Sports Sci. 2008;26(14):1557–65. Epub 2008/10/25. 10.1080/02640410802334196 . 18949660

[23] Cooper AR, Andersen LB, Wedderkopp N, Page AS, Froberg K. Physical activity levels of children who walk, cycle, or are driven to school. Am J Prev Med. 2005;29(3):179–84. 10.1016/j.amepre.2005.05.009 . 16168866

[24] Eime RM, Young JA, Harvey JT, Charity MJ, Payne WR. A systematic review of the psychological and social benefits of participation in sport for children and adolescents: informing development of a conceptual model of health through sport. The international journal of behavioral nutrition and physical activity. 2013;10:98 Epub 2013/08/16. 10.1186/1479-5868-10-98 23945179 PMC3751802

[25] Basterfield L, Reilly JK, Pearce MS, Parkinson KN, Adamson AJ, Reilly JJ, et al Longitudinal associations between sports participation, body composition and physical activity from childhood to adolescence. J Sci Med Sport. 2015;18(2):178–82. Epub 2014/04/08. 10.1016/j.jsams.2014.03.005 . 24704422 PMC4364369

[26] Gomersall SR, Rowlands AV, English C, Maher C, Olds TS. The ActivityStat hypothesis: the concept, the evidence and the methodologies. Sports medicine. 2013;43(2):135–49. Epub 2013/01/19. 10.1007/s40279-012-0008-7 . 23329607

[27] Gomersall S, Maher C, Norton K, Dollman J, Tomkinson G, Esterman A, et al Testing the activitystat hypothesis: a randomised controlled trial protocol. BMC Public Health. 2012;12:851 Epub 2012/10/10. 10.1186/1471-2458-12-851 23043381 PMC3503831

[28] Joy EA. Practical approaches to office-based physical activity promotion for children and adolescents. Curr Sports Med Rep. 2008;7(6):367–72. Epub 2008/11/14. 10.1249/JSR.0b013e31818ec87b . 19005361

[29] Sallis R. Developing healthcare systems to support exercise: exercise as the fifth vital sign. Br J Sports Med. 2011;45(6):473–4. Epub 2011/02/05. 10.1136/bjsm.2010.083469 . 21292925

[30] Lobelo F, Stoutenberg M, Hutber A. The Exercise is Medicine Global Health Initiative: a 2014 update. Br J Sports Med. 2014;48(22):1627–33. 10.1136/bjsports-2013-093080 Epub 2014 Apr 23. Epub 2014/04/25 . 24759911

